# Circ_0110940 Exerts an Antiapoptotic and Pro-Proliferative Effect in Gastric Cancer Cells via the miR-1178-3p/SLC38A6 Axis

**DOI:** 10.1155/2022/3494057

**Published:** 2022-06-30

**Authors:** Xiaonan Miao, Haiou Zou, Lijuan Pan, Jing Cheng, Youshan Wu, Rui Chen, Yong Su, Hongwei Du

**Affiliations:** ^1^Department of Gastroenterology, Lianyungang Oriental Hospital, The Affiliated Lianyungang Oriental Hospital of Xuzhou Medical University, The Affiliated Lianyungang Oriental Hospital of Kangda College of Nanjing Medical University, Lianyungang 222000, Jiangsu, China; ^2^Department of Clinical Laboratory, Affiliated Hospital of Yangzhou University, Yangzhou 225001, Jiangsu, China; ^3^Department of Gastroenterology Medicine, Lanzhou Second People's Hospital, Lanzhou 730046, Gansu, China

## Abstract

Circular RNAs (circRNAs) are essential regulators in human cancers, including gastric cancer, by the miRNA/mRNA axis. A previous study identified the upregulation of circ_0110940 in human gastric cancer tissues. The present study performed *in vitro* assays to reveal the functions of circ_0110940 and its downstream miRNA/mRNA axis in gastric cancer cells. Traditional proliferation and apoptosis assays including colony formation, EdU staining, and Annexin V-PI staining assays were conducted. A luciferase reporter assay was performed to assess the binding between miR-1178-3p and circ_0110940 or SLC38A. We found the significant upregulation of circ_0110940 in human gastric cancer cells AGS and MKN45. Circ_0110940 was a stable circRNA and exerted an antiproliferative and proapoptotic effect in AGS and MKN45. Circ_0110940 binded with miR-1178-3p, which further targeted SLC38A6 3′UTR. Circ_0110940 degraded miR-1178-3p, and miR-1178-3p degraded SLC38A6. Thus, circ_0110940 has a positive effect on SLC38A6 expression. Furthermore, SLC38A6 rescued the effects of circ_0110940 knockdown on gastric cancer cell proliferation and apoptosis. In conclusion, circ_0110940 exerted an antiapoptotic and pro-proliferative effect in gastric cancer cells via the miR-1178-3p/SLC38A6 axis, which may provide basis for the targeted therapy of gastric cancer.

## 1. Introduction

Cancer is a malignant disease and causes a great burden on the global population. The traditional methods for treating cancer consist of surgery, radiotherapy, and chemotherapy. New treatments including targeted therapy, immunotherapy, cryoablation, and hormone therapy have been developed [1–4]. Targeted therapy has a unique strength because of its specificity to cancer cells while avoiding causing toxicity to off-target cells [2]. Gastric cancer is the sixth most common cancer with new cases of 1,089,103 and the second leading cause of cancer death with new deaths of 768,793 worldwide in 2020 [5]. Three drugs including trastuzumab [6], ramucirumab [7], and pembrolizumab [8] are currently approved by the FDA to treat gastric cancer. These drugs have good effects on breast cancer and colon cancer. However, they are not effective enough for treating gastric cancer. Thus, to explore novel, effective targeted therapy for gastric cancer is needed.

Circular RNAs (circRNAs) are endogenous biomolecules that are regulated by specific cis-acting elements and trans-acting factors. These covalently closed biomolecules are expressed in specific cells and tissues in eukaryotes. Compared with linear RNAs, circRNAs lack 5′ and 3′ ends and are resistant to RNA exonuclease, which makes them serve as promising biomarkers and therapeutic targets. Advances in circRNA-specific computational tools and high-throughput RNA sequencing have promoted the detection, quantification, and functional characterization of circRNAs [9]. Many circRNAs act as microRNA (miRNA) inhibitors (“sponges”) to exert their regulatory functions and are implicated in cancers including gastric cancer by modulating miRNAs. Circ_0008259, derived from LMO7 pre-mRNA, is highly expressed in gastric cancer, promoting the proliferation, motility, and regulating the glutamine metabolism of gastric cancer cells by “sponging” miR-30a-3p [10]. High expression of circ-RanGAP1 has a significant association with lymph node metastasis, advanced TNM stage, and worse survival in patients with gastric cancer and contributes to gastric cancer progression dependent on the miR-877-3p/VEGFA pathway [11]. Epstein–Barr virus-encoded circLMP2A induces and maintains stemness phenotypes in gastric cancer by inhibiting miR-3908 [12].

According to a previous study by Wang et al. [13], circRNA located at chr1: 1572769–1635783 (annotated as circ_0110940 by circBase [14]) showed the most significantly abnormal expression in gastric cancer tissues compared with para-gastric cancer tissues (log_2_FC = 6.99, *P*=2.35°Ε^−06^). Circ_0110940 is formed by back-splicing of the exons 2–10 of CDK11B pre-mRNA. According to the circBase annotation, circ_0110940 has a genomic length of 63014 and a spliced length of 48427. Despite the fact that there are some studies demonstrating the association of CDK11B and tumorigenesis [15, 16], no published papers have revealed the role of circ_0110940. We detected expression of circ_0110940 in gastric cancer cells and identified its upregulation. Next, the functions of circ_0110940 and its downstream miRNA/mRNA axis in gastric cancer cells were investigated.

## 2. Material and Method

### 2.1. Cell Culture

Two human gastric cancer cell lines, MKN-45 (CL-0292, Procell) and AGS (CL-0022, Procell), and a normal stomach mucosa epithelium cell line GES-1 (CL-0563, Procell) were used in this study. AGS was cultured in F12K medium, and MKN-45 and GES-1 were cultured in RPMI 1640 medium. The culture atmosphere is 37°C, 90% humidity, and 5% CO_2_. Both media were added with 10% fetal bovine serum (C0251, Beyotime) and 1% penicillin/streptomycin (ST488, Beyotime).

### 2.2. Oligonucleotide Transfection

MKN-45 and AGS were seeded in a 6-well plate for incubation overnight. Scrambled shRNA negative control, shRNA targeting circ_0110940, pcDNA3.1 (^+^) vector expressing circ_0110940 or SLC38A6, miR-1178-3p mimics, and NC mimics were all commercially provided by GenePharma, Shanghai, China, and were transfected into MKN-45 and AGS using Lipofectamine RNAiMax (Life Technologies) based on the manufacturer's protocols.

### 2.3. Reverse Transcription-Quantitative PCR (RT-qPCR)

TRIzol (Invitrogen) was used for total RNA extraction from GES-1, MKN-45, and AGS cells. 500 ng of total RNA at a volume of 10 *μ*l was used for reverse transcription using the PrimeScript RT Master Mix (RR036A; Takara, Dalian, China). qPCR was performed with the TB Green® Premix Ex Taq (RR420A; Takara). Thermocycling conditions were set as follows: initial denaturation at 95°C for 30 sec, 40 cycles at 95°C for 5 sec and 60°C for 30 sec, and extension at 65–95°C, in 0.5°C increments for 5 sec. RNA expression was quantified with the 2^−ΔΔCq^ method [17] and was normalized to GAPDH (for circ_0110940 and SLC38A6) or U6 (for miR-1178-3p). The items used for amplification were listed as follows:

circ_0110940, F, 5′-TCAAGAGCCTGATGGAGACC-3′ and *R*, 5′-AGCAGGTTGGACGTCTTGAG-3'; CDK11B, F, 5′-AATTGTTGCGTCTAATGCCAT-3′ and *R*, 5′-GACGCTTCGGTAATTAAAAATGTC-3'; SLC38A6, F, 5′-AATGCCATCATGGGAAGTG-3′ and *R*, 5′-GCAGCAAGAAGCTAAATCCA-3'; GAPDH, F, 5′-GAAGATCAAGATCATTGCTCCTC-3′ and *R*, 5′-ATCCACATCTGCTGGAAGG-3'; miR-1178-3p, F, 5′-TTGCTCACTGTTCTTCCCTAGC-3′ and *R*, 5′-CTCTACAGCTATATTGCCAGCCAC-3'; U6, F, 5′-ATACAGAGAAAGTTAGCACGG-3′ and *R*, 5′-GGAATGCTTCAAAGAGTTGTG-3'.

### 2.4. RNase *R* Digestion Test

The total RNA of gastric cancer cells was extracted and divided into two parts. One part was digested: 2.5 *μ*g total RNA was added with 3 U RNase *R* and incubated at 37°C for 20 min; another part was added with 0.5 *μ*L DEPC water as a control. After the reaction, RNA reverse transcription and PCR amplification were performed to detect the expression level of RNA.

### 2.5. Actinomycin *D* Test

The logarithmic gastric cancer cells were inoculated into 6-well plates with 7 × 10^4^ cells per well and cultured for 24 h. Then, fresh complete medium containing 2 *μ*g/ml actinomycin *D* was added. Cell RNA was extracted at 1 h, 2 h, 6 h, 8 h, and 10 h after treatment, and the circRNA and mRNA abundance were observed after actinomycin *D* treatment by RT-qPCR.

### 2.6. Western Blotting

After lysing using the RIPA Lysis Buffer (Beyotime), samples were separated on SDS-PAGE gels and transferred to PVDF membranes (Millipore). Membranes were processed using a BeyoECL Plus Ultrasensitive ECL Chemiluminescence Kit following the manufacturer's protocol (Beyotime). The following primary antibodies from Abcam (Shanghai, China) were used: anti-Bax (1 : 1000; ab32503), anticleaved caspase-3 (1 : 500; ab32042), anticaspase-3 (1 : 5000; ab32351), and anti-GAPDH (1 : 2500; ab9485). HRP-labeled anti-IgG was used as the secondary antibody. GAPDH is a loading control.

### 2.7. Proliferation Detection

A colony formation assay and an EdU staining assay were used to assess cell proliferation. For the colony formation assay, cells were seeded in a 6-cm plate at a density of 500/well and cultured in a complete culture medium for two weeks. After discarding the culture solution, cells were fixed with methanol for 10 min, treated with crystal violet dye for 15 min, washed with running water, and dried. Colonies (>50 cells) were finally photographed and counted. For the EdU staining assay, cells were seeded into 96-well plates at a density of 6,000 cells/well and transfected for 24 h. Cells were incubated with the EdU reagent A for 2 h in an incubator at 37°C. Following the manufacturer's instructions of a Cell-Light™ EdU Apollo643 *In Vitro* kit (C10310-2, RiboBio, Guangzhou), subsequent experimental steps were performed.

### 2.8. Annexin V-PI Staining

AGS and MKN45 cells were seeded in a 6-well plate at a concentration of 5.0 × 10^4^ cells/well and transfected for 24 h. Cells were centrifugated and resuspended in 195 *μ*L Annexin V-FITC binding solution. Next, the cells were stained with 5 *μ*L Annexin V-FITC and 10 *μ*L PI using an apoptosis detection kit (C1062S, Beyotime) in the dark at room temperature for half an hour. Annexin^+^PI^+^ and Annexin^+^PI^−^ cells were considered as apoptotic cells. Cells were analyzed using a Beckman Cyan flow cytometer on the CellQuest 7.1 software (Beckman).

### 2.9. Luciferase Reporter Assay

MiR-1178-3p sequences (5′-UUGCUCACUGUUCUUCCCUAG-3′) and fragments of SLC38A6 3′UTR (5′-CCAACCUCCAGAACUGUGAGCAA-3′) were synthesized by GenePharma and subcloned into pmirGLO vectors (Promega). PmirGLO-miR-1178-3p was cotransfected with pcDNA-circ_0110940, while pmirGLO-SLC38A6 3′UTR was cotransfected with miR-1178-3p mimics into AGS and MKN45 cells. 24 h later after transfection, luciferase activity was determined with a Dual Luciferase Reporter Assay kit (Promega) and was calculated as the ratio of firefly luciferase activity to *Renilla* luciferase activity.

### 2.10. Statistical Analysis

*P* values were derived by the Student's *t*-test and one/two-way analysis of variance using the GraphPad prism tool. Any value reported in graphs is an average of three independent experiments, and error bars represented SD. *P* < 0.05 indicates statistical significance.

## 3. Result

### 3.1. Validation of the Circular Characteristics of circ_0110940

**Figure 1:**
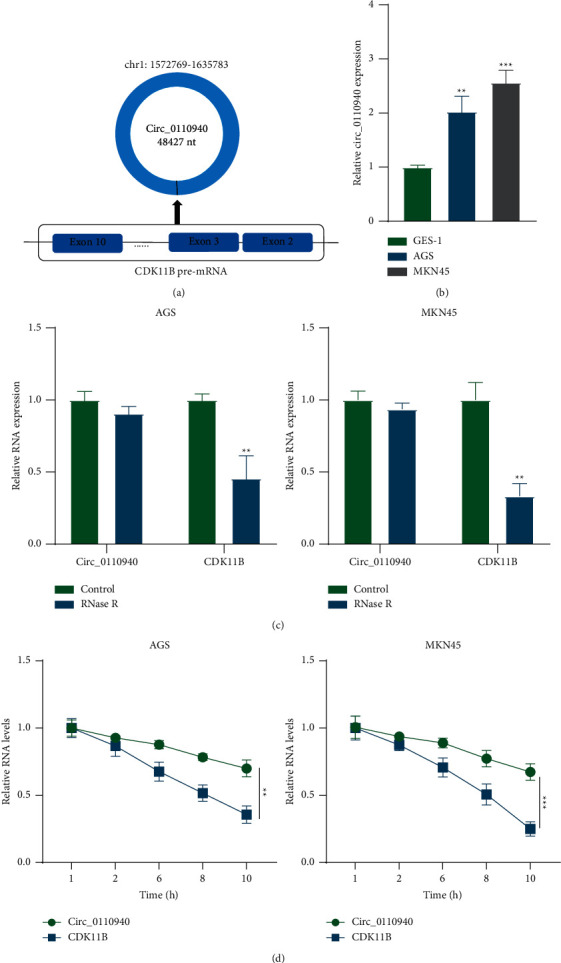
Validation of the circular characteristics of circ_0110940. (a) A schematic map briefly revealed the formation of circ_0110940. (b) Circ_0110940 expression in MKN45 and AGS cells compared to GES-1 was assessed by RT-qPCR. (c) Circ_0110940 and CDK11B expression in MKN45 and AGS cells after treatment of RNase R (3U Rnase R/*μ*g RNA) for 20 min at 37°C was detected by RT-qPCR. (d) Circ_0110940 and CDK11B expression after treatment of 20 *μ*M actinomycin D for 1, 2, 6, 8, and 10 h was detected by RT-qPCR. ^*∗∗*^*P* < 0.01, ^*∗∗∗*^*P* < 0.001.

Circ_0110940 is formed by back-splicing of the exons 2–10 in the CDK11B pre-mRNA and is annotated by circBase to have a genomic length of 63014 and a spliced length of 48427 (Figure 1(a)). It showed significant upregulation in AGS and MKN45 cells compared with the control GES-1 cell line (Figure 1(b)). The results of the RNase *R* exonuclease assay using RT-qPCR revealed that circ_0110940 was resistant to digestion, while CDK11B was not resistant to digestion, indicating the circular nature of circ_0110940 (Figure 1(c)). Moreover, circ_0110940 is more resistant to actinomycin *D* than CDK11B, further demonstrating the circular characteristics of circ_0110940 (Figure 1(d)).

### 3.2. Circ_0110940 Knockdown Exerts a Proapoptotic and Antiproliferative Effect in AGS and MKN45 Cells

**Figure 2:**
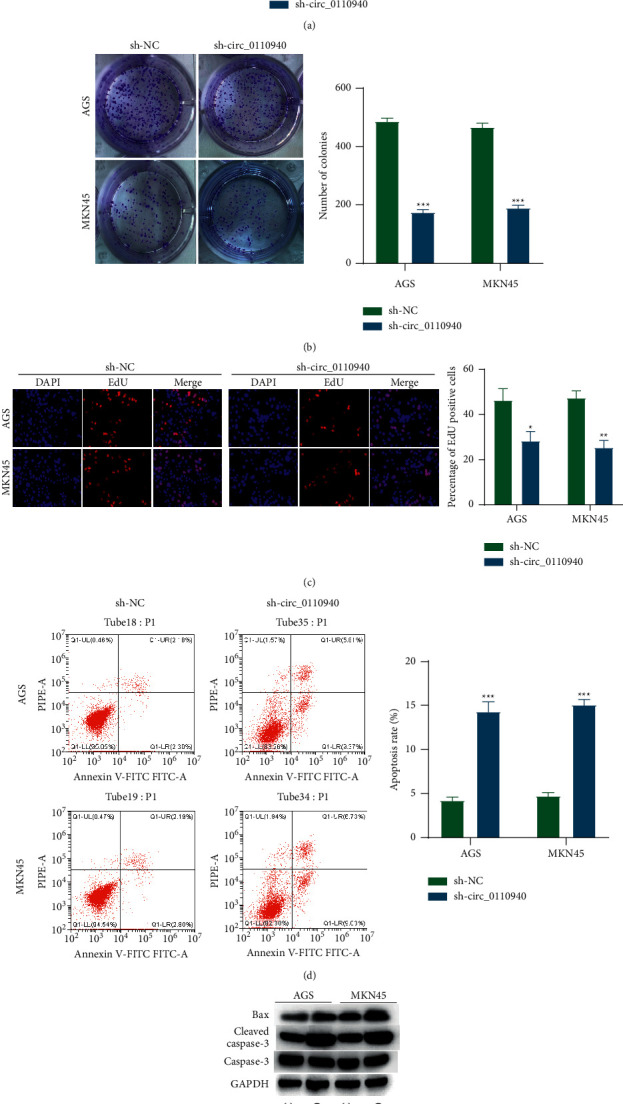
Circ_0110940 exerts a proapoptotic and antiproliferative effect in AGS and MKN45 cells. (a) RT-qPCR analysis of circ_0110940 expression after transfection with sh-circ_0110940 for 24 h. (b) Number of colonies formed by MKN45 and AGS cells under transfection of sh-circ_0110940. (c) Percentage of EdU-positive cells under the condition of circ_0110940 knockdown. (d) Annexin + PI + and Annexin + PI- cells under the condition of circ_0110940 knockdown were analyzed using flow cytometry. (e) Protein bands of Bax, cleaved caspase-3, and caspase-3. ^*∗*^*P* < 0.05, ^*∗∗*^*P* < 0.01, ^*∗∗∗*^*P* < 0.001.

We used sh-circ_0110940 targeting circ_0110940 to knockdown circ_0110940 in AGS and MKN45 cells, and RT-qPCR verified the knockdown efficiency of circ_0110940 (Figure 2(a)). Sh-circ_0110940 reduced the number of colonies that were formed by AGS and MKN45 cells and decreased the percentage of EdU-positive cells, indicating that silenced circ_0110940 had a negative effect on gastric cancer cell proliferation (Figures 2(b) and 2(c)). Sh-circ_0110940 increased the percentage of Annexin^+^PI^+^ and Annexin^+^PI^−^ cells, revealing the promotive influence of silenced circ_0110940 on gastric cancer cell apoptosis (Figure 2(d)). Moreover, two apoptosis indexes, Bax and cleaved caspase-3, were detected using Western blotting. Sh-circ_0110940 increased the protein expression of Bax and cleaved caspase-3 (Figure 2(e)).

### 3.3. Circ_0110940 Binds with miR-1178-3p to Suppress Its Expression

**Figure 3:**
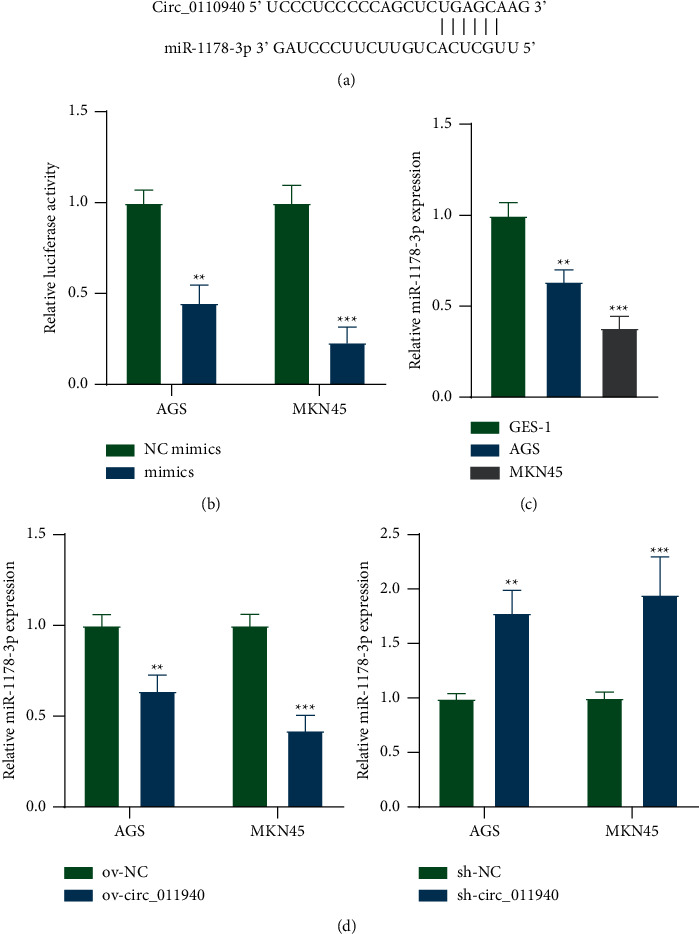
Circ_0110940 binds with miR-1178-3p to suppress its expression. (a) Binding site of miR-1178-3p on position 2262–2268 of circ_0110940 was obtained from the Circular RNA Interactome database. (b) Relative luciferase activity of pmirGLO-miR-1178-3p in MKN45 and AGS cells that overexpressed circ_0110940. (c) RT-qPCR analysis of miR-1178-3p expression in MKN45 and AGS cells compared to GES-1. (d) Relative miR-1178-3p expression in MKN45 and AGS cells that overexpressed or silenced circ_0110940 was measured by RT-qPCR. ^*∗∗*^*P* < 0.01, ^*∗∗∗*^*P* < 0.001.

The circular RNA Interactome database [18] was used to predict the miRNAs that bind to circ_0110940. MiR-1178-3p was identified to bind to circ_0110940 in four sites (positions: 2262 to 2268; 24879 to 24886; 36019 to 36025; 45186 to 45192). Binding sequences of miR-1178-3p on positions 2262–2268 in circ_0110940 were revealed in Figure 3(a). A luciferase reporter assay was performed and verified the binding of miR-1178-3p on UGAGCA in circ_0110940 (Figure 3(b)). MiR-1178-3p expression exhibited downregulation in AGS and MKN45 cells (Figure 3(c)). Transfection of ov-circ_0110940 reduced expression of miR-1178-3p, while sh-circ_0110940 promoted miR-1178-3p level in AGS and MKN45 cells (Figure 3(d)).

### 3.4. MiR-1178-3p Negatively Regulates SLC38A6 Expression

**Figure 4:**
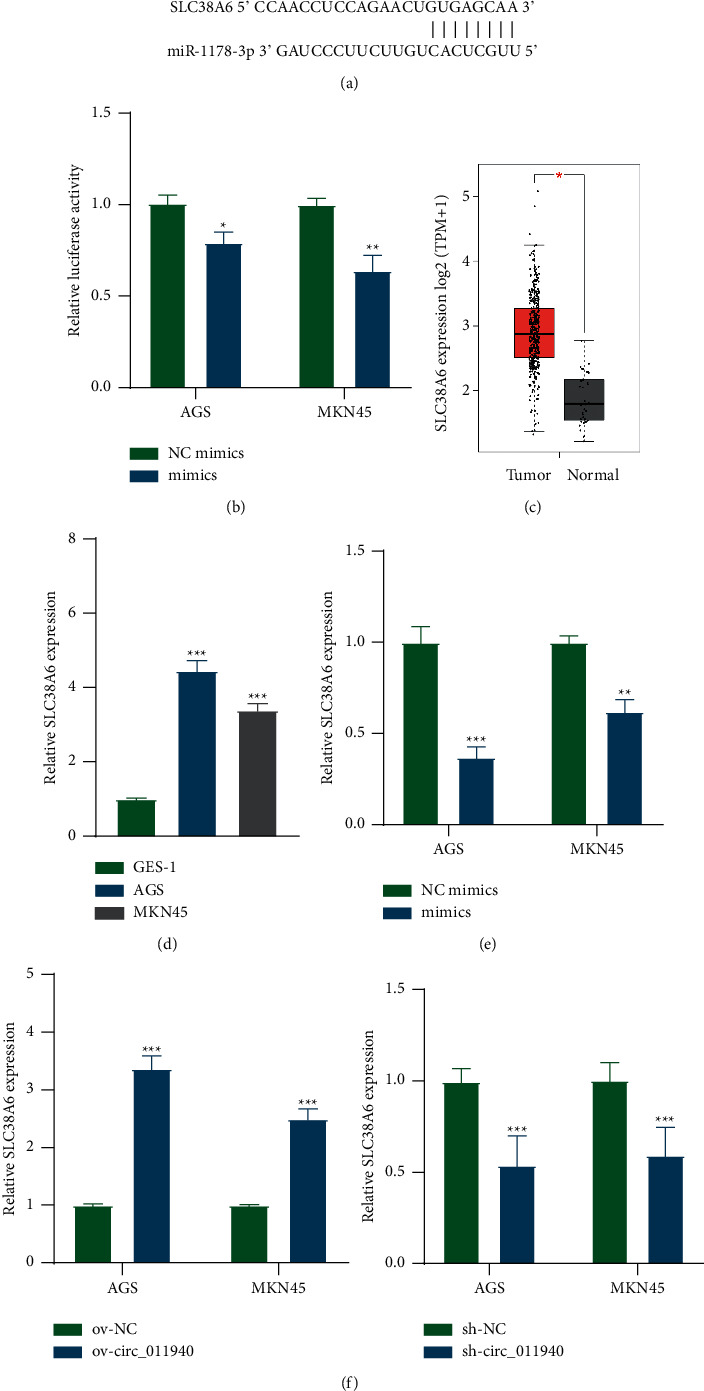
MiR-1178-3p negatively regulates SLC38A6 expression. (a) Binding site of miR-1178-3p on position 27–34 of SLC38A6 3′UTR was obtained from TargetScan. (b) Relative luciferase activity of pmirGLO-SLC38A6 3′UTR in MKN45 and AGS cells that overexpressed miR-1178-3p. (c) Upregulation of SLC38A6 in 408 stomach adenocarcinoma tissues compared with 36 normal tissues was obtained from the GEPIA database. (d) RT-qPCR analysis of SLC38A6 expression in MKN45 and AGS cells compared to GES-1. (e-f) Relative SLC38A6 expression in MKN45 and AGS cells was measured by RT-qPCR. ^*∗*^*P* < 0.05, ^*∗∗*^*P* < 0.01, ^*∗∗∗*^*P* < 0.001.

Targets of miR-1178-3p were searched using the Target scan database [19]. SLC38A6 showed the highest binding potential and was verified in the present study. Binding sequences of miR-1178-3p on position 27–34 of SLC38A6 3′UTR are revealed in Figures 4(a)and 4(b) indicated that miR-1178-3p degraded SLC38A6 3′UTR. SLC38A6 is revealed to be upregulated in 408 stomach adenocarcinoma tissues compared with 36 normal tissues based on GEPIA database [20] (Figure 4(c)). We further revealed the upregulation of SLC38A6 in AGS and MKN45 gastric cancer cells (Figure 4(d)). MiR-1178-3p exerted a negative effect on SLC38A6 mRNA expression (Figure 4(e)). After overexpression of circ_0110940 , SLC38A6 level was enhanced; whereas knockdown of circ_0110940 hindered SLC38A6 expression in AGS and MKN45 cells (Figure 4(f)).

### 3.5. SLC38A6 Rescued the Effects of Silenced circ_0110940 on Gastric Cancer Cell Proliferation and Apoptosis

**Figure 5:**
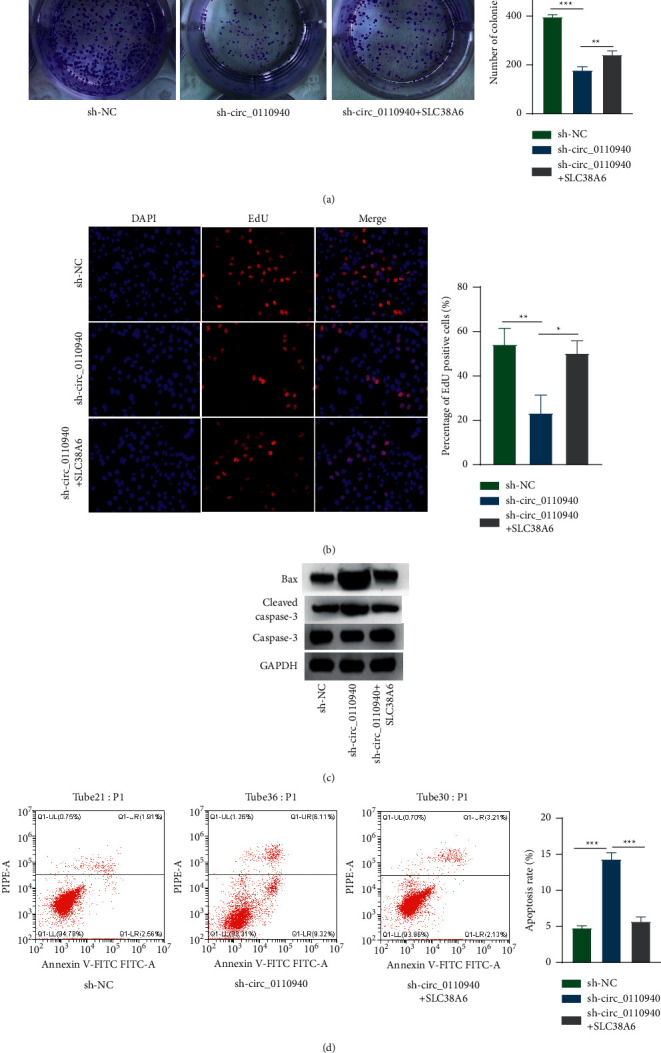
SLC38A6 rescued the effects of silenced circ_0110940 on gastric cancer cell proliferation and apoptosis. (a) Number of colonies formed by MKN45 cells under transfection of sh-circ_0110940 or cotransfection of sh-circ_0110940 + ov-SLC38A6. (b) Percentage of EdU-positive cells. (c) Western blotting of Bax, cleaved caspase-3, and caspase-3 proteins. (d) Annexin + PI + and Annexin + PI- cells were analyzed by flow cytometry. ^*∗*^*P* < 0.05, ^*∗∗*^*P* < 0.01, ^*∗∗∗*^*P* < 0.001.

Finally, we cotransfected sh-circ_0110940 and ov-SLC38A6 in MKN45 cells. As shown in Figure 5(a)–5(d), silenced circ_0110940 presented antiproliferative and proapoptotic effect in MKN cells. However, the influence on MKN45 cell proliferation and apoptosis induced by sh-circ_0110940 should be partially counterbalanced by cotransfection with ov-SLC38A6. Collectively, the above-mentioned results indicated that circ_0110940 modulated the proliferation and apoptosis of gastric cancer cells by upregulating SLC38A6 (Figure 5(a)–5(d)).

## 4. Discussion

Several circRNAs participate in the modulation of gastric cancer cell biological activities [10–13]. In this study, we focused on circ_0110940 in gastric cancer cells. Circ_0110940 is formed by back-splicing of the exons 2–10 in the CDK11B pre-mRNA. CDK11 B is a member of CDKs that significantly mediate tumor cell proliferation and growth [21]. CDK11 B has the potential to promote the self-renewal capability of hepatocellular carcinoma stem cells and enhance their oncogenicity *in vivo* [16] and is associated with the poor prognosis of colon cancer [15]. After the validation of the circular feature of circ_0110940, its functions were evaluated. It was found that shRNA-mediated circ_0110940 knockdown exerts a proapoptotic and antiproliferative effect in AGS and MKN45 cells, indicating the oncogenic role of circ_0110940 in gastric cancer. Moreover, the downstream axis of circ_0110940 was searched using bioinformatics analysis and verified by experiments.

MiR-1178-3p was identified to bind to circ_0110940 and showed downregulation in gastric cancer cells, which indicated that miR-1178-3p is an antioncogene in gastric cancer. However, several studies have demonstrated the tumor promoter role of miR-1178-3p in cancers. MiR-1178-3p has been reported to be “sponged” by circFNDC3B in bladder cancer and contributes to malignant phenotypes of bladder cancer cells [22]. MiR-1178-3p exerts an oncogenic role to facilitate nasopharyngeal carcinoma cell proliferation and motility by binding to STK4 [23]. MiR-1178-3p is “sponged” by hsa_circ_0077837 and rescued its suppressive effects on non-small-cell lung cancer cell proliferation, viability, and motility [24]. On the contrary, miR-1178-3p regulates the PI3K/Akt pathway by targeting TBL1XR1 to suppress hepatocellular carcinoma cell growth *in vitro* and inhibit xenograft tumor growth *in vivo* [25].

Next, we found that miR-1178-3p potentially targets SLC38A6 based on bioinformatics analysis. As a member of the SLC38 family, SLC38A6 is selectively expressed in the excitatory neurons of the brain [26] and is deemed as an orphan transporter with an unknown substrate profile [27]. A previous study revealed the upregulation of SLC38A6 in pancreatic adenocarcinoma [28]. In our study, upregulation of SLC38A6 in gastric cancer cells was verified after we identified that it exhibited higher expression in gastric cancer tissues than control tissues based on bioinformatics analysis. SLC38A6 serves as a direct target of miR-1178-3p in gastric cancer cells. MiR-1178-3p can cause SLC38A6 degradation by binding to the 3′UTR of SLC38A6. Considering that circ_0110940 can degrade miR-1178-3p, positive regulation of circ_0110940 on SLC38A6 was obvious.

However, some limitations of this study must be addressed. First, we lacked an *in vivo* assay to verify the oncogenic role of circ_0110940. Second, expression of circ_0110940/miR-1178-3p/SLC38A6 in human gastric cancer tissues needs to be validated. Moreover, since gastric cancer exhibits high metastasis, the effects of circ_0110940/miR-1178-3p/SLC38A6 on cell migration and invasion deserve further exploration.

In conclusion, circ_0110940 promoted cell proliferative ability and inhibited cell apoptotic capacity in gastric cancer cells via the miR-1178-3p/SLC38A6 axis, which may provide a basis for the targeted therapy of gastric cancer.

## Data Availability

All data generated or analyzed during this study are available from the corresponding author upon reasonable request.
